# Reconsidering Trust and Information Engagement and Unpacking the Role of Emotion in Public Responses During the Early Stage of a Public Health Crisis in China: Web-Based Survey Study

**DOI:** 10.2196/77790

**Published:** 2025-08-25

**Authors:** Zhiming Liu, Jiawei Tu, Tien-Tsung Lee, Lu Wei

**Affiliations:** 1 Department of Communication Faculty of Social Sciences University of Macau Taipa, Macau China; 2 Communication University of Zhejiang Hangzhou China; 3 College of Media and International Culture Zhejiang University Hangzhou China

**Keywords:** risk communication, trust, negative emotions, information engagement, public health crisis

## Abstract

**Background:**

The COVID-19 pandemic continues to offer valuable insights into crisis management and risk communication, particularly through retrospective analyses that allow a more comprehensive understanding. Emotional responses played a crucial role in shaping how individuals processed information and built trust in different objects in the early stages of the COVID-19 pandemic.

**Objective:**

This study aimed to investigate how negative emotions influence online information engagement and trust in 4 distinct entities: government, scientists, health care providers, and other people (relatives, friends, family, and strangers).

**Methods:**

A nationwide survey was conducted in China from January 31 to February 9, 2020, involving 1568 adult participants. The data collection was particularly valuable due to the limited access to national samples in China during the early stages of the public health crisis. Participants were asked questions related to negative emotions, engagement with online information, and their trust in 4 different entities (government, scientists, other people, and health care providers) during the pandemic. Mediation analyses were performed to test the associations between the examined variables. A 95% bootstrap CI approach was used to estimate the mediation effects.

**Results:**

This study reveals that negative emotions not only had a direct effect on trust but also indirectly fostered trust in the government and scientists through increased information engagement. There was a positive association (B=0.219, SE 0.023; *P*<.001) between negative emotions and information engagement. In addition, individuals experiencing more negative emotions tended to trust more in the government (B=0.191, SE 0.022; *P*<.001) and scientists (B=0.184, SE 0.017; *P*<.001). However, this effect did not extend to trust in health care providers or interpersonal trust.

**Conclusions:**

The research findings reveal that while negative emotions directly and indirectly enhanced trust in the government and scientists through increased information engagement, they did not significantly impact trust in health care providers or interpersonal relationships in the Chinese context. These findings highlight the different pathways through which emotions and information behaviors affect trust during public health crises, offering critical lessons for future public health emergencies and risk communication.

## Introduction

### Background

The COVID-19 pandemic, spanning from its initial outbreak in December 2019 to its gradual containment, has left an indelible mark all over the world in various aspects of human lives [1,2]. A substantial body of research has continued to revisit the impacts, evaluate preventive strategies, and reflect on the effectiveness of risk communication approaches [3,4].

During the onset of the COVID-19 outbreak in China, the populace grappled with heightened negative emotions triggered by the uncertainties and challenges posed by the deadly virus [5,6]. Quarantine measures confined individuals to their homes, and their access to information and interaction with others was primarily online [7,8]. Such isolation not only altered how people obtained and used information but also influenced their perceptions of societal dynamics at large, fostering a climate characterized by such negative emotions as fear and anxiety [6,9].

While facing a highly contagious and deadly virus, people had to make critical decisions on whom and what to trust [3,10,11]. Thus, investigating how to foster trust is inevitable for risk communication and crisis management [1,3,12]. While previous studies have investigated the role of emotions and information use in a pandemic [5,9], this study, drawing upon the risk information seeking and processing (RISP) model [13], delves into the complex interplay between psychological states, information engagement behaviors, and their potential effects on trust toward different and significant entities including the government, scientists, health care providers, and other people to analyze the factors that influence trust in the initial outbreak of a crisis in China.

### Literature Review

#### Trust and Its Consequences

Trust can be defined as the conviction (how) in the reliability of another person (who) regarding a specific issue (what) that arises in the face of uncertainties [14-16]. Trust plays a pivotal role in society, particularly in risk communication and crisis management, as it directly shapes behavioral compliance during times of uncertainty [17]. Public trust serves as a critical factor in facilitating or undermining cooperative intentions toward authorities while also sustaining confidence in institutional structures, governance systems, and response mechanisms [3,12]. Therefore, understanding the mechanisms and conditions under which trust is formed or eroded is essential for improving crisis response, designing more resilient communication strategies, and promoting behavioral compliance for future emergencies.

Research has revealed that trust can lead to cooperative beliefs and behaviors when faced with uncertainties [18,19]. Survey studies uncovered a positive connection between trust and social distancing and self-care behavior [3,10]. In addition an online survey from 11 countries found that people’s evaluation of the severity of the COVID-19 pandemic was influenced by their trust in the news media and their perception of social media bias [18]. During the COVID-19 pandemic in the United States, individuals who trusted right-leaning more than left-leaning media engaged in notably fewer preventive actions [19].

However, how trust in different entities develops and functions during the early stages of a public crisis varies across social settings and leads to diverse understandings. China—widely discussed in crisis management yet understudied regarding its social trust for the emerging phase of crisis when fear and ignorance prevail—presents both theoretical and practical significance for this investigation.

#### Types and Objects of Trust

Factors such as different forms of information resources can influence *types* and *objects* of trust [20-22]. The object of trust can be another person, a group of people, an organization, an institution, a geopolitical region, or the entire world [23]. Two types of trust, *interpersonal* and *impersonal* trust are conceptualized differently [14,24,25]. *Interpersonal* trust which can be defined as trust in other persons, like familiar people like family members is important [26,27]. *Interpersonal* trust can shape people’s inclinations to action, particularly during a crisis or a pandemic [28,29]. *Impersonal* trust is also known as institutional [30-32] or systems trust [20,33,34]. It can be characterized as the expectation or confidence that social systems or institutions would act morally and uphold norms [27]. People’s attitudes toward health-related policies issued by social institutions (eg, the government) or systems (eg, health care authorities) largely depend on public trust in the entities that enact the policies [35-37].

Types of trust take on a different meaning and inclinations in a Chinese setting. The Chinese tend to have a higher level of trust in institutions and organizations, such as the government or authoritative figures such as scientists [37-40]. Research reveals that a high level of trust in authorities and experts can facilitate the acceptance of preventive measures, reduce panic, and enhance the effectiveness of risk communication strategies [35,36]. Conversely, distrust can lead to misinformation, resistance, and harmful behaviors [41]. However, the Chinese have a comparatively lower level of trust in interpersonal relationships, particularly with strangers, which hinders interpersonal cooperation as well as health-related behavior, particularly social distancing [41,42]. Both scientists (health experts) and health care providers can play a vital role in communicating health-related knowledge and information in everyday life [41,42]. However, when the public is confronted with a health crisis, the dynamics of trust in scientists versus health care providers can vary [43]. For instance, physicians who directly treat the public and offer suggestions are generally more trusted by Americans than scientists in related fields [44,45].

#### Emotions, Risk Information Seeking, and Trust

The RISP model provides a theoretical framework for understanding how individuals respond to crises and risks through cognitive (systematic) and affective (heuristic) processes [13]. It emphasizes that in the face of uncertainty, people engage in information-seeking and processing behaviors influenced by factors such as emotions, perceived risk, informational insufficiency, and social norms [46,47]. This study builds upon and extends the RISP model, examining how negative emotions influence different objects of trust through information-seeking behavior during an emerging public health crisis.

The *psychological demand* (emotions) for information sufficiency serves as the primary driver behind the proactive search and methodological processing of risk information in the RISP model [48]. Research indicates that information-seeking and avoidance can be motivated by both positive and negative emotions [49-52]. Scholars revealed that people with positive emotions tend to have a holistic perception of seeking information more comprehensively during a pandemic [46].

Negative emotions, such as fear and anxiety, are common psychological reactions when facing a crisis such as the Middle East respiratory syndrome, H1N1 influenza, and H7N9 influenza [10,53,54]. In the early stage of the COVID-19 pandemic, the preventive measures imposed by the government exacerbated prevalent negative emotions such as frustration, hostility, and anger [55,56]. Previous research has examined the relationship between emotions and information use behavior, for instance, Bohner and Weinerth [57] demonstrated that while positive emotion reduces people’s tendency to examine information, negative affect enhances it. Park et al [47] found that people’s emotional reactions, such as worry and fear, were triggered by their perceived risk, and this exacerbated the perception of their own lack of information.

We suspect that negative emotions are positively associated with information engagement. Therefore, we formulated this hypothesis:

- H1: Negative emotions have a positive relationship with information engagement at the early stage of a pandemic.

The RISP model posits that affective responses and information insufficiency might enhance information-seeking and engaging behaviors that are related to one’s trust [48,58]. Previous studies have investigated the impact of negative emotions, such as anger, sadness, and anxiety, on trust during public health crises [10,59,60]. A line of studies found that negative emotions decrease trust [61-63]. However, Zhang et al [64] found that negative emotions—particularly anger—could increase trust. Researchers have also examined the relationships between emotions, information seeking or engagement, and trust, particularly regarding trust in government. For example, Ahn et al [65] made cross-cultural comparisons and concluded that early in the COVID-19 pandemic in South Korea, the United States, and Singapore, trust in the government was negatively related to and reduced by negative emotions (anger, fear, sadness, and anxiety), and positively related to hope. In addition, these emotions acted as a mediator in the relationship between trust and information seeking. Erhardt et al [66] discovered that negative emotions have an impact on public trust in the government during times of crisis. For instance, anger places the blame for unfavorable circumstances on the government. Khalifa [67] reported that among the Bahrainis, trust in the government during the COVID-19 pandemic was positively connected with the use of news websites, but negatively associated with dependency on television and social media. On the basis of the aforementioned theoretical frameworks and findings, this research aims to dive into the relationships between negative emotions, information engagement, and trust in the different key objects during the early stage of the COVID-19 pandemic in China.

Because previous research has no consensus on the relationship between negative emotions, information engagement behavior, and trust, this study chose to propose the following 2 research questions (RQs):

- RQ1: How are negative emotions associated with trust in different objects?
- RQ2: How does trust in different objects relate to negative emotions and information engagement?

In our data, we have 4 different but significant objects of trust: the government, scientists, others (interpersonal trust), and health care providers. How each is operationalized is explained in the Methods section.

**Figure 1:**
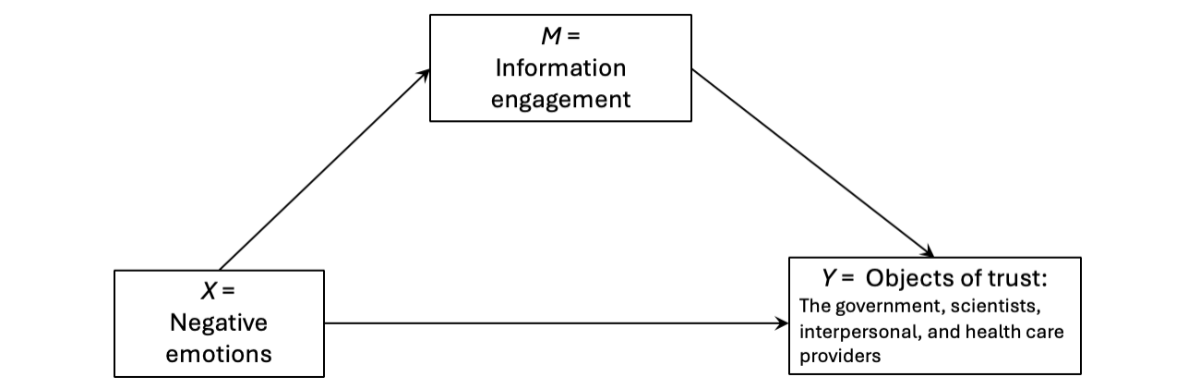
The proposed model of the relationships between variables.

To summarize the assumptions of the relationships between negative emotions, information seeking, and trust in various entities, Figure 1 is the conceptual model that theorizes the relationships between these 3 variables. This study examines the potential mediating roles of media use, drawing on the media practice model, which conceptualizes media engagement and its outcomes as part of a reciprocal, cyclical process rather than a straightforward linear effect [68,69]. Specifically, we suspected that information engagement mediates the association between negative emotions and trust. Therefore, we created 4 mediation models, one with each object of trust, to be tested.

## Methods

### Data

An online survey was conducted during the early stage of the COVID-19 outbreak in China, from January 31, 2020, to February 9, 2020. The data collection was particularly valuable due to the limited access to national samples in China during the early stages of the public health crisis. Sojump, a professional Chinese internet survey company that is similar to SurveyMonkey, gathered the data. It first randomly selected 2840 people from its sample pool and then sent them an email invitation to participate. A total of 1656 people completed the survey, with a response rate of 58.3%. This sampling technique was a time-efficient way to investigate public emotions and opinions during the COVID-19 outbreak. Previous research conducted in China has taken this approach [70-72].

Participants who completed the survey (a questionnaire with 179 items) in fewer than 11 minutes [73] or failed the attention checks were excluded, ending with 1568 valid cases. A total of 31 provinces, municipalities, and autonomous territories in mainland China were included in the sample. With a mean age of 31.02 (SD 9.0), there were 789 (50.32%) male respondents and 779 (49.68%) female respondents. Furthermore, 18.11% (284/1568) of the participants had a monthly household income of ≤CN ¥5000; 31.12% (488/1568) of the participants earned between CN ¥5001 and CN ¥10,000; 21.17% (332/1568) of the participants earned between CN ¥10,001 and CN ¥15,000; 15.75% (247/1568) of the participants earned between CN ¥15,001 and CN ¥20,000; 12.4% (194/1568) of the participants earned between 20,001 and 50,000 CN ¥; and 1.47% (23/1568) of the participants earned >CN ¥50,000 CN ¥. On a 1-to-10-point scale of income, the mean is 6.29 (SD 1.96), which is in the category of CN ¥8001 to CN ¥10,000. Only 5.04% (79/1568) of the sample had completed high school or less, 16.07% (252/1568) were current or former technical school students, 69.07% (1083/1568) were college students or alumni, and 9.82% (154/1568) were postgraduates. On a 1 to 9-point scale of education, the mean was 6.76 (SD .92), which falls into the category of some or professional college.

### Ethical Considerations

The instrument and data collection received approval from the Institutional Review Board of Zhejiang University (2020-056). Prior to completing the online survey, all participants provided voluntary informed consent. Each respondent received a compensation of CN¥ 12 (US $1.60) for their participation. The dataset was fully anonymized, with no personally identifiable information retained or linked to any participant.

### Measurements

The dependent variables (*Y*) measure one’s trust in four distinct entities: (1) the local government in terms of its response to the pandemic (Cronbach α=0.85); (2) scientists studying and battling the virus (Cronbach α=0.79); (3) interpersonal actors (family, relatives, friends, and strangers regarding the disclosure of their health conditions and travel histories; Cronbach α=0.66); and (4) the health care providers who treated COVID-19 infections (Cronbach α=0.75).

In the initial stage of a crisis, individuals often experience a constellation of negative emotions [47,74]. To capture the overall emotional response during the early stage of the public health crisis, the independent variable (*X*) is a combination of 5 negative emotions—fear, anxiety, anger, frustration, and hostility (Cronbach α=0.79). This approach follows prior research that conceptualizes emotional arousal as a general affective state influencing information processing and behavioral intention [10,53]. The mediator (*M*) is a scale of how often people engaged with different information-related activities in the context of COVID-19, such as posting an original message or liking, commenting on, or forwarding or sharing messages that they had encountered (Cronbach α=0.72).

Similar research has studied the same variables [75-78]. Control variables are typical demographics in mass and health communication research, including sex, age, income, and education [79]. Table 1 summarizes these variables. The PROCESS Macro (Model 4, version 4.1) proposed by Hayes [80] for SPSS was used to analyze the data, with 5000 bootstrap samples. Continuous variables were mean-centered before analysis. We created a mediation model for each of the 4 objects of trust. Only models with statistical significance are reported.

**Table 1:** A summary of dependent, independent, and control variables.

Variables and wording				Mean (SD)		Cronbach α
**Dependent variable 1: trust in the government^a^**						.85
	**How do you rate the performance of the local government of where you live? (average scale)**		3.90 (0.70)			
		1. I have confidence in the ability of local government departments in terms of controlling the pandemic.	4.02 (0.79)			
		2. Relevant local government departments will fully consider the benefits of the people in the context of the pandemic.	3.85 (0.89)			
		3. Relevant local government departments show fairness when dealing with the pandemic.	3.80 (0.91)			
		4. Relevant local government departments are transparent when conveying information about the pandemic.	3.80 (1.0)			
		5. Overall, relevant local government departments are trustworthy in their handling of the pandemic.	4.03 (0.84)			
**Dependent variable 2: trust in scientists^a^**						.79
	**How do you rate the scientists who are on the front line fighting COVID-19? (average scale)**		4.35 (0.56)			
		1. They deserve my trust.	4.36 (0.64)			
		2. Their professional qualifications are trustworthy.	4.38 (0.70)			
		3. They put people’s health as a topic priority.	4.30 (0.80)			
		4. I trust them.	4.37 (0.71)			
**Dependent variable 3: trust–interpersonal^b^**						.66
	**When you ask the following people about their recent travel histories and health conditions, how much do you trust their answers? (average scale)**		3.63 (0.55)			
		1. Family	4.53 (0.71)			
		2. Relatives	3.80 (0.84)			
		3. Friends	3.85 (0.75)			
		4. Strangers	2.35 (0.81)			
**Dependent variable 4: trust in health care providers^a^**						.75
	**How much do you agree with the following statements? (average scale)**		3.51 (0.75)			
		1. The confirmation rates of COVID-19 in hospitals are low (reverse-coded).	3.91 (0.76)			
		2. Hospitals can treat patients effectively because they are overcrowded and have a shortage of supplies (reverse-coded).	2.94 (1.11)			
		3. Physicians do not have enough knowledge about COVID-19 and are prone to misdiagnosis (reverse-coded).	3.93 (0.95)			
		4. Hospitals are prone to misdiagnosis because they do not have enough COVID-19 test kits (reverse-coded).	3.32 (1.19)			
		5. Hospitals cannot effectively isolate COVID-19 patients, which will likely cause cross-contamination (reverse-coded).	3.45 (1.22)			
***X*: negative emotions^a^**						.79
	**The country is extremely concerned about the current COVID-19 pandemic. How much do you exhibit or feel each of the following emotions during this outbreak? (average scale)**		3.73 (0.82)			
		1. Fear	2.91 (1.03)			
		2. Anxiety	2.97 (1.16)			
		3. Anger	2.43 (1.27)			
		4. Frustration	2.16 (1.03)			
		5. Hostility	1.69 (0.96)			
***M*: engagement with information^c^**						.72
	**When you are exposed to online information related to the COVID-19 pandemic, how do you respond to the information? (average scale)**		2.85 (0.77)			
		1. Give a like	2.83 (2.20)			
		2. Post original message	1.91 (1.07)			
		3. Comment	2.68 (1.14)			
		4. Forward or reshare	3.11 (1.18)			
		5. Search further for related information	3.75 (1.03)			
**Control 1**						
	Age (range 16 to 67 years)		31.02 (9.0)		—^d^	
**Control 2**						
	Sex (male: 50.3%; female: 49.7%); dummy-coded male		—		—	
**Control 3**						
	Education (1-9 scale; 1=no schooling; 9=doctorate)		6.76 (0.92)		—	
**Control 4**						
	Monthly income (1-10 scale; 1=no income; 10= ≥CN ¥50,001 ^e^)		6.29 (1.96)		—	

^a^1=totally disagree, 2=disagree, 3=neither disagree nor agree, 4=agree, and 5=totally agree.

^b^1=totally trust, 7=totally distrust.

^c^1=none, 2=selfdom, 3=several times, 4=often, and 5=very frequently.

^d^Not available.

^e^CN ¥1=US $0.14.

## Results

The first hypothesis posits that the more negative emotions one has, the more they are likely to engage with information. The mediation model shown in Table 2 indicates that there was a positive association (B=0.219, SE 0.023; *P*<.001) between negative emotions and information engagement. Therefore, H1 was supported.

RQ1 asks about the relationship between negative emotions and trust in different objects. Higher levels of negative emotions were linked to *more* trust in the government, scientists, and interpersonal trust, but *less* trust in health care providers.

RQ2 asks whether information engagement has a mediating effect on the path between negative emotions and trust in 4 different entities. The answer to RQ2 is that information engagement had a mediating effect between negative emotions and trust in the government and scientists, but *not* in health care providers or interpersonal trust.

**Figure 2:**
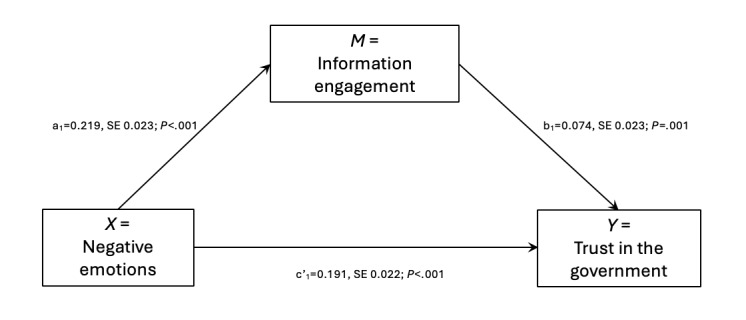
The mediating role of information engagement in the relationship between negative emotions and government trust.

The trust-in-the-government mediation model was supported, as Table 2 and Figure 2 demonstrate. The a_1_-path from negative emotion to information engagement (*X* predicting *M*) was significant (B=0.219, SE 0.023; *P*<.001), suggesting that individuals experiencing more negative emotions such as fear, anger, and anxiety tended to be more information-engaging. There was also a significant and positive coefficient (B=0.074, SE 0.023; *P*=.001) for the b_1_-path from information engagement to trust in the government (*M* predicting *Y*). Also notable was the c’₁-path coefficient (X predicting Y), which reflects the direct effect of negative emotions on trust in the government (B=0.191, SE 0.022; *P*<.001). The total effect from *X* (negative emotions) to *Y* (trust in the government) was 0.207, SE 0.021; *P*<.001. The indirect effect from *X* (negative emotions) to *Y* (trust in the government) was 0.016, SE 0.01, 95% CI 0.01-0.027. Because 0.01 and 0.027 are both on the same side of zero, this indirect impact was validated. This set of findings suggests that an individual’s trust in the government was positively associated with their level of negative emotions. In addition, experiencing *more* negative emotions encouraged people to engage with *more* information, which *raised* their level of trust in the government.

**Figure 3:**
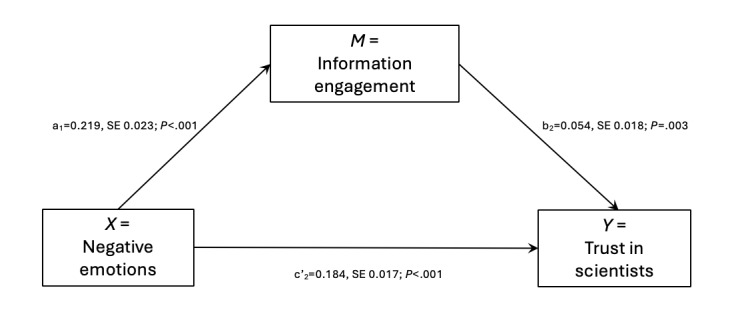
The mediating role of information engagement in the relationship between negative emotions and trust in scientists.

Figure 3 and the data in Table 2 show that the trust-in-the-scientists mediation model stood. Evidence of the connection between negative emotions and information engagement (*X* predicting *M*) is found in the a_2_-path (B=0.219, SE 0.023; *P*<.001). The b_2_-path from information engagement to trust in scientists (*M* predicting *Y*) had a significant and positive coefficient (B=0.054, SE 0.018; *P*=.003). Another significant relationship between negative emotions and trust in scientists was the c’_2_-path (*X* predicting *Y*) coefficient (B=0.184, SE 0.017; *P*=.003). The total effect from *X* (negative emotions) to *Y* (trust in scientists) was 0.196; SE 0.017; *P*<.001. The indirect effect from *X* (negative emotions) to *Y* (trust in scientists) was 0.012; SE 0.01; 95% CI 0.004-0.02. Because 0.004 and 0.02 are both on the same side of zero, the indirect effect through mediation was confirmed. This implies that a person’s degree of negative emotions rose with their degree of trust in scientists. Furthermore, feeling *more* negative emotions motivated people to engage with *more* information, hence *increasing* their level of trust in scientists.

**Table 2:** Trust in the government and scientists regression models generated by PROCESS Model 4.

Variables			B (SE; 95% CI)		*P* value
**Predicting information engagement^a^**					
	Negative emotions	0.219 (0.023; 0.174 to 0.265)		<.001	
	Age (years)	−0.006 (0.002; −0.010 to –0.001)		.02	
	Gender	0.006 (0.038; −0.069 to 0.080)		.88	
	Education	0.038 (0.022; −0.006 to 0.081)		.09	
	Income	0.045 (0.012; 0.024 to 0.066)		<.001	
**Predicting trust in the government^b^**					
	Negative emotions	0.191 (0.022; 0.148 to 0.233)		<.001	
	Information engagement	0.074 (0.023; 0.029 to 0.120)		.001	
	Age (years)	0.005 (0.002; 0.001 to 0.010)		.001	
	Gender	0.024 (0.035; −0.044 to 0.092)		.49	
	Education	−0.026 (0.020; −0.066 to 0.014)		.21	
	Income	0.015 (0.010; −0.005 to 0.035)		.13	
**Predicting trust in scientists^c^**					
	Negative emotions	0.184 (0.017; 0.150 to 0.218)		<.001	
	Information engagement	0.054 (0.018; 0.018 to 0.090)		.003	
	Age (years)	−0.002 (0.002; −0.005 to 0.001)		.26	
	Gender	−0.009 (0.027; −0.062 to 0.045)		.75	
	Education	−0.009 (0.016; −0.040 to 0.022)		.57	
	Income	−0.008 (0.008; −0.023 to 0.007)		.29	

^a^*R*^2^=0.070; *F*_5,1562_=23.46.

^b^*R*^2^=0.073; *F*_6,1561_=20.41.

^c^*R*^2^=0.089; *F*_6,1561_=25.38.

## Discussion

### Principal Findings

This research reveals that individuals’ negative emotions are both directly and indirectly (through information engagement) positively associated with their trust in government and scientists. However, these negative emotions do not affect respondents’ trust in others (relatives, friends, family, and strangers) or health care providers. These discrepancies with previous findings [65] call for further investigation into factors that influence public responses during health crises and risks, particularly in the initial stages of a public health emergency [47].

### The Objects of Trust

Although negative emotions tend to encourage a higher level of engagement with information, echoing previous findings [46,57,65], the consequent effect on trust relies heavily on the specific entities involved. The fact that negative emotions and information engagement are positively associated with trust in the government and scientists, but have no connection with trust in others (interpersonal trust) or health care providers, underscores the necessity for a more intricate understanding of the interplay between the dynamics of trust.

As argued earlier, the objects of trust can be roughly divided into 2 camps: persons versus institutions or systems [14,24,30]. The analysis reveals that the negative emotions and information engagement are positively associated with trust toward *institutional* or *impersonal* entities in our models, namely the government and scientists who supposedly possessed more knowledge about how to combat the deadly virus. These 2 entities are not individual “everyday people” and they tend not to have direct contact with the general population. The government is associated with formal roles and responsibilities that impact the broader community yet may not have direct personal interactions with most individuals. Scientists were the authoritative heroes in a far way laboratory figuring out how to battle the virus. So both the government and scientists were *impersonal* to our survey participants, and they were the ones that our survey respondents likely looked up to and trusted.

In addition, trust in government in the Chinese context is multilayered and dynamic, often differing across levels (central vs local) and shaped by both political culture and past governance performance [81]. Previous studies suggest that local governments in China typically receive less trust than central authorities, particularly during uncertain times [82]. However, this study examines how local government trust and digital engagement interact during unique circumstances where negative emotions can drive information-seeking behavior that enhances—rather than weakens—institutional trust.

Conversely, findings suggest that during the early stage of the COVID-19 pandemic in China, individuals’ negative emotions and information engagement were not associated with their trust in the types of persons that they had *direct and tangible contact* with in their daily lives. Family members and friends are within everyday social interactions and health care providers are the ones in the hospital who can be reached when necessary. COVID-19 is a contagious virus that spreads among people and interpersonal trust in China has been decreasing [83]. Any person could be a virus carrier or transmitter. People either did not know whether they had the virus or had an incentive to lie about their health conditions or travel histories so that they would not be quarantined. Therefore, other people were less likely to be trusted in this context, leading to an insignificant outcome in our analysis. In addition, during the initial stage of this pandemic, health care providers likely knew about the virus and how to treat infections as little as ordinary citizens. Therefore, trust in health care providers is not a significant factor in our findings.

### The Effects of Information Engagement

In the initial stage of the COVID-19 pandemic in China, the pervasive sense of fear, anger, and anxiety likely triggered a heightened state of negative emotions among the populace. Faced with a novel and rapidly evolving crisis, individuals probably sought out information that could provide them with a sense of security, reassurance, and guidance. Selective and avoidance exposure originated from the theory of cognitive dissonance, which holds that people experience discomfort or dissonance when they are exposed to facts or situations that contradict their personal beliefs [84,85]. Selective exposure—a psychological strategy used to reduce feelings of dissonance—involves deliberately avoiding information that contradicts one’s views and seeking out information that supports existing beliefs.

When the public is faced with uncertainty and lacks the competence to make decisions, trust in scientists plays a crucial role in science-related decisions [27,86]. Following the COVID-19 pandemic outbreak, particularly after January 20, 2020, when Zhong Nanshan, a highly regarded Chinese expert was sent to Wuhan by the National Health Commission to investigate the situation and confirm the human-to-human transmission of SARS-CoV-2, a unique form of collaboration between the government and representative scientists emerged [87]. The government created a communication matrix for scientists, and scientists acted as vital microphones for the government to inform the public of important information [87,88]. The communication matrix accompanied by the highly centralized media system made Chinese news media the major information source. The media focused on the government’s and scientists’ responses to the pandemic, highlighting preemptive steps and decisive acts, which could increase public confidence in their capabilities [89]. Consequently, the government and scientists, as primary sources of information and guidance during the crisis, gained prominence in the media and public discourse [11,40,90].

Individuals experiencing heightened negative emotions were likely to selectively expose themselves to information disseminated by trusted governmental and scientific authorities. The lack of direct personal involvement with these authoritative figures might have contributed to a more idealized perception of their capabilities and intentions. In contrast, during the early stage of the pandemic, the public knew that hospitals were overwhelmed, leading to the likely perception that health care providers could not handle the situation adequately.

### Sociocultural Factors

While earlier studies, notably Ahn et al [65], suggested a *negative* correlation between negative emotions and trust, our study revealed a *positive* relationship, namely negative emotions *fostering* an increase in trust toward governmental authorities and scientists.

Cultural frameworks contribute to explaining variations in belief and behavior patterns across nations. China is collectivist with a greater power distance [91-93]. In other words, Chinese culture emphasizes collective harmony and societal order. Therefore, Chinese people—in comparison with citizens in individualist cultures—would respect and trust the government as well as authority figures such as scientists more [40,94], particularly in uncertain times. To preserve societal stability and cohesiveness, the cultural tendency of deference to authority and hierarchy may encourage people to believe in the government and scientists, particularly in the face of negative emotions.

Moreover, a greater sense of dependence on government-led initiatives and outlets occurred among the Chinese during times of crisis due to its highly centralized governmental structure, and a tradition of information control [95,96]. At the early stage of a public health crisis, even in the face of unpleasant emotional experiences, citizens may view the government and scientific authorities as important sources of direction and assistance, particularly when facing difficulties.

### Implications

This study offers important theoretical contributions to the fields of risk communication and crisis management. The RISP model [13,48], which highlights the role of emotions in shaping trust and information engagement during crises, provides the foundation for this research, which extends the literature by examining how negative emotions differentially influence trust toward various entities, including the government, scientists, health care providers, and interpersonal networks. By demonstrating that negative emotions primarily fostered trust in governmental and scientific institutions but not in health care providers or social relations, the findings underscore the need for a more nuanced understanding of trust formation processes. Moreover, this study emphasizes the importance of incorporating sociocultural factors into analyses of emotion-trust dynamics. The findings contradict previous studies [65] showing that revealed negative emotions and trust in the government are typically negatively associated in the face of a crisis. This research calls for a more nuanced, context-sensitive approach in theorizing about emotion, information engagement, and trust during public health crises.

Practically, the findings of this study offer valuable insights for future risk management and communication strategies during public health emergencies. Understanding that negative emotions can selectively reinforce trust in certain institutions highlights the need for targeted communication efforts that recognize the differential credibility assigned to various sources [12]. Risk communicators and policy makers should design emotion-sensitive strategies that not only acknowledge public emotional states but also actively guide information engagement toward credible institutions to strengthen societal resilience [1]. Furthermore, by recognizing the influence of sociocultural contexts on trust dynamics, practitioners can better tailor their interventions to the specific cultural and institutional environments in which crises unfold [65]. These lessons provide a critical foundation for developing more effective, culturally adaptive risk communication frameworks that can better manage uncertainty, foster trust, and promote collective action in the face of future crises.

### Limitations and Suggestions for Future Research

This study has several limitations that should be acknowledged. First, the sample is not fully representative of the broader population, and the data collection period is relatively brief. With 69.07% (1083/1568) of the respondents being current university students or graduates, and 9.82% (154/1568) being postgraduates, highly educated people are overrepresented. This skewness is a common attribute observed in online nonprobability survey samples, potentially limiting the generalizability of the findings [97]. Nevertheless, given the unique circumstances of the crisis’s initial outbreak and infeasibility to access the representative sample, this method represented the most effective approach for the data collection. Second, while the RISP model includes key constructs such as perceived risk, this variable was not incorporated into our model. Instead, this study focused on how negative emotions influence trust in different entities, offering a theoretical extension by emphasizing the object-specific nature of trust. In addition, because this research gathered data in China, its findings may have limited generalizability to other populations.

Notwithstanding these limitations, we believe this research has yielded valuable knowledge. We have learned about how people deal with emotions, information engagement, and trust at the earliest stage of a national or global crisis. This timing provided a unique opportunity to examine the Chinese public’s trust dynamics and emotional responses during crisis management at an early stage when uncertainty was at its peak and society faced unprecedented unknowns.

Future research should use a more representative survey sample and ask questions about trust across different levels of the government as well as the use of additional types of information sources. Including open-ended questions would help reveal why respondents trust or distrust specific entities. In addition, researchers should investigate distinct dimensions of interpersonal trust—between close ties and strangers—to develop a more nuanced understanding of its nature.

### Conclusions

This study revisited the early stage of the COVID-19 pandemic in China to examine the complex interplay between negative emotions, information engagement, and trust in different actors. The findings revealed that while negative emotions directly and indirectly enhanced trust in the government and scientists through increased information engagement, they did not significantly impact trust in health care providers or interpersonal relationships. This study has contributed to a deeper understanding of the Chinese context, in comparison with other countries and cultures. The observed positive relationship between negative emotions, information engagement, and trust in the government and authoritative scientists likely signifies a distinctive pattern specific to the Chinese sociocultural context, diverging from the trends identified in previous studies conducted in different national settings, such as in the United States, Korea, Singapore [65], and Bahrain [67]. In addition, there is an observed difference between scientists and health care providers as trustees in the face of a pandemic. These noteworthy distinctions underscore the influence of unique cultural and societal norms when the chips are down in shaping individuals’ perceptions and responses, highlighting the importance of contextual factors in shaping trust dynamics within diverse sociocultural landscapes.

This study offers valuable lessons for designing future strategies for public health crisis and risk communication. Recognizing the selective nature of trust formation and tailoring communication efforts to emotional and cultural contexts can enhance the effectiveness of crisis response. As the world continues to face emerging public health threats, this type of retrospective analysis provides vital insights for developing more resilient and effective public health communication systems.

## Abbreviations

RISP: risk information seeking and processing
RQ: research question

## References

[1] Coombs WT (2018). Ongoing Crisis Communication: Planning, Managing, and Responding.

[2] Flor LS, Friedman J, Spencer CN, Cagney J, Arrieta A, Herbert ME, Stein C, Mullany EC, Hon J, Patwardhan V, Barber RM, Collins JK, Hay SI, Lim SS, Lozano R, Mokdad AH, Murray CJL, Reiner RC, Sorensen RJ, Haakenstad A, Pigott DM, Gakidou E (2022). Quantifying the effects of the COVID-19 pandemic on gender equality on health, social, and economic indicators: a comprehensive review of data from March, 2020, to September, 2021. Lancet.

[3] Liu Z, Tu J, Lee TT, Wei L (2025). Does trust affect behavior in a public health crisis? Testing an extended theory of planned behavior model with trust. J Conting Crisis Manage.

[4] de Macedo Steffen G, Lacerda DP, Richter C, de Souza Gomes RF (2025). Leading adaptive risk context: contingent plans of Covid-19 at universities. Int J Disaster Risk Reduct.

[5] Li Y, Zeng Y, Liu G, Lu D, Yang H, Ying Z, Hu Y, Qiu J, Zhang C, Fall K, Fang F, Valdimarsdóttir UA, Zhang W, Song H (2020). Public awareness, emotional reactions and human mobility in response to the COVID-19 outbreak in China – a population-based ecological study. Psychol Med.

[6] Su Y, Wu P, Li S, Xue J, Zhu T (2020). Public emotion responses during COVID-19 in China on social media: an observational study. Hum Behav Emerg Technol.

[7] Xie X, Zang Z, Ponzoa JM (2020). The information impact of network media, the psychological reaction to the COVID-19 pandemic, and online knowledge acquisition: evidence from Chinese college students. J Innov Knowl.

[8] Zhao Y, Xu S, Wang L, Huang Y, Xu Y, Xu Y, Lv Q, Wang Z, Wu Q (2020). Concerns about information regarding COVID-19 on the internet: cross-sectional study. J Med Internet Res.

[9] Dong W, Tao J, Xia X, Ye L, Xu H, Jiang P, Liu Y (2020). Public emotions and rumors spread during the COVID-19 epidemic in China: web-based correlation study. J Med Internet Res.

[10] Wei L, Huang Q (2024). Retrospecting digital media use, negative emotions, and trust gaps during the COVID-19 pandemic in China: cross-sectional web-based survey. J Med Internet Res.

[11] Guo D, Liu S, Sun Y (2025). Who can help me? Citizens' help-seeking on Weibo during the Shanghai lockdown. Disaster Med Public Health Prep.

[12] Boin A, McConnell A, Hart P (2021). Governing the Pandemic: The Politics of Navigating a Mega-Crisis.

[13] Shah Z, Wei L (2022). Interpersonal risk communication matters more than media risk communication in its impact on individuals' trust and preventive behaviors during COVID-19. Int J Disaster Risk Reduct.

[14] Hardin R (1993). The street-level epistemology of trust. Politics Soc.

[15] Robbins BG (2016). What is trust? A multidisciplinary review, critique, and synthesis. Sociol Compass.

[16] Rotter JB (1971). Generalized expectancies for interpersonal trust. Am Psychol.

[17] Tsfati Y, Cappella JN (2003). Do people watch what they do not trust?: exploring the association between news media skepticism and exposure. Commun Res.

[18] Han R, Xu J, Pan D (2022). How media exposure, media trust, and media bias perception influence public evaluation of COVID-19 pandemic in international metropolises. Int J Environ Res Public Health.

[19] Zhao E, Wu Q, Crimmins EM, Ailshire JA (2020). Media trust and infection mitigating behaviours during the COVID-19 pandemic in the USA. BMJ Glob Health.

[20] Blöbaum B (2016). Key factors in the process of trust. On the analysis of trust under digital conditions. Trust and Communication in a Digitized World.

[21] van der Meer TW, Ouattara E (2019). Putting ‘political’ back in political trust: an IRT test of the unidimensionality and cross-national equivalence of political trust measures. Qual Quant.

[22] Zmerli S, Newton K, Zmerli S, van der Meer TW (2017). Objects of political and social trust: scales and hierarchies. Handbook on Political Trust.

[23] Alon I, Bar-Tal D (2017). The Role of Trust in Conflict Resolution: The Israeli-Palestinian Case and Beyond.

[24] Levi M, Braithwaite V, Levi M (1998). A state of trust. Trust and Governance.

[25] Safari A, Barzoki AS, Heidari Aqagoli P (2020). Exploring the antecedents and consequences of impersonal trust. Int J Organ Anal.

[26] Flanagan CA, Stout M (2010). Developmental patterns of social trust between early and late adolescence: age and school climate effects. J Res Adolesc.

[27] Yuan H, Long Q, Huang G, Huang L, Luo S (2022). Different roles of interpersonal trust and institutional trust in COVID-19 pandemic control. Soc Sci Med.

[28] Raven J, Wurie H, Witter S (2018). Health workers' experiences of coping with the Ebola epidemic in Sierra Leone's health system: a qualitative study. BMC Health Serv Res.

[29] Stefaniak A, Wohl MJ, Elgar FJ (2022). Commentary on "Different roles of interpersonal trust and institutional trust in COVID-19 pandemic control". Soc Sci Med.

[30] Domański H, Pokropek A (2021). The relation between interpersonal and institutional trust in European countries: which came first?. Polish Sociol Rev.

[31] Letki N (2006). Investigating the roots of civic morality: trust, social capital, and institutional performance. Polit Behav.

[32] Offe K, Warren ME (1999). How can we trust our fellow citizens?. Democracy and Trust.

[33] Luhmann N, Davis H, Raffan J, Rooney K, King M, Morgner C (2017). Trust and Power.

[34] Vanhala M, Puumalainen K, Blomqvist K (2011). Impersonal trust: the development of the construct and the scale. Pers Rev.

[35] Salmon DA, Dudley MZ, Glanz JM, Omer SB (2015). Vaccine hesitancy: causes, consequences, and a call to action. Vaccine.

[36] Schmelz K (2021). Enforcement may crowd out voluntary support for COVID-19 policies, especially where trust in government is weak and in a liberal society. Proc Natl Acad Sci U S A.

[37] Gilson L (2003). Trust and the development of health care as a social institution. Soc Sci Med.

[38] Torpe L, Lolle H (2010). Identifying social trust in cross-country analysis: do we really measure the same?. Soc Indic Res.

[39] Wei L, Yan C (2018). Social quality of China: indicators, reality, and problems. Int J Soc Quality.

[40] Liu SN, Chang TK (2016). One disaster, three institutional responses: legitimation crisis and competing discourses in China. Journal Stud.

[41] Bursztyn L, Rao A, Roth CP, Yanagizawa-Drott DH (2020). Misinformation during a pandemic. National Bureau of Economic Research.

[42] Yang F, Huang Z (2021). Health communication and trust in institutions during the COVID-19 lockdown in China's urban communities. Urban Governance.

[43] Algan Y, Cohen D, Davoine E, Foucault M, Stantcheva S (2021). Trust in scientists in times of pandemic: panel evidence from 12 countries. Proc Natl Acad Sci U S A.

[44] Funk C, Hefferon M, Kennedy B, Johnson C (2019). Trust and mistrust in Americans’ views of scientific experts. Pew Research Center.

[45] Leonard MB, Pursley DM, Robinson LA, Abman SH, Davis JM (2022). The importance of trustworthiness: lessons from the COVID-19 pandemic. Pediatr Res.

[46] Huang J, Ren W, Wang S, Zhou Y, Yang Y (2023). Positive emotion and media dependence: measuring risk information seeking and perception in the COVID-19 pandemic prevention. Inquiry.

[47] Park T, Ju I, Ohs JE, Hinsley A, Muzumdar J (2023). Information seeking during the COVID-19 pandemic: application of the risk information seeking and processing model. Res Social Adm Pharm.

[48] Griffin RJ, Dunwoody S, Neuwirth K (1999). Proposed model of the relationship of risk information seeking and processing to the development of preventive behaviors. Environ Res.

[49] de los Santos TM, Nabi RL (2019). Emotionally charged: exploring the role of emotion in online news information seeking and processing. J Broadcast Electron Media.

[50] Khan SS (2017). Developing positive emotion through affective design for interactive information seeking. Proceedings of the Seventh BCS-IRSG Symposium on Future Directions in Information Access.

[51] Li J, Zheng H (2020). Online information seeking and disease prevention intent during COVID-19 outbreak. Journal Mass Commun Q.

[52] Lyons J, Sokhey A (2014). Emotion, motivation, and social information seeking about politics. Polit Commun.

[53] Chen X, Huang B, Lin W (2023). The effect of interpersonal relationship and epidemic attention on negative emotion among medical students: the mediating role of social satisfaction. BMC Psychiatry.

[54] Hao R, Liu Y, Shen W, Zhao R, Jiang B, Song H, Yan M, Ma H (2022). Surveillance of emerging infectious diseases for biosecurity. Sci China Life Sci.

[55] Han S, Sun T, Sun Y, Gao X (2023). Public psychological crisis reports: an investigation during the remission of COVID-19 in China. Chin Manag Stud.

[56] Dubey S, Biswas P, Ghosh R, Chatterjee S, Dubey MJ, Chatterjee S, Lahiri D, Lavie CJ (2020). Psychosocial impact of COVID-19. Diabetes Metab Syndr.

[57] Bohner G, Weinerth T (2001). Negative affect can increase or decrease message scrutiny: the affect interpretation hypothesis. Pers Soc Psychol Bull.

[58] So J, Kuang K, Cho H (2016). Information seeking upon exposure to risk messages: predictors, outcomes, and mediating roles of health information seeking. Commun Res.

[59] Folkman S, Moskowitz JT, Ozer EM, Park CL, Gottlieb BH (1997). Positive meaningful events and coping in the context of HIV/AIDS. Coping with Chronic Stress.

[60] Vemprala N, Bhatt P, Valecha R, Rao HR (2021). Emotions during the COVID-19 crisis: a health versus economy analysis of public responses. Am Behav Sci.

[61] Rodriguez-Sanchez C, Schuitema G, Claudy M, Sancho-Esper F (2018). How trust and emotions influence policy acceptance: the case of the Irish water charges. Br J Soc Psychol.

[62] Smith N, Leiserowitz A (2014). The role of emotion in global warming policy support and opposition. Risk Anal.

[63] Yuan Y, Yang S, Jiang X, Sun X, Lin Y, Liu Z, Zhu Y, Zhao Q (2022). Trust in government buffers the negative effect of rumor exposure on people's emotions. Curr Psychol.

[64] Zhang K, Goetz T, Chen F, Sverdlik A (2021). Angry women are more trusting: the differential effects of perceived social distance on trust behavior. Front Psychol.

[65] Ahn J, Kim HK, Kahlor LA, Atkinson L, Noh GY (2021). The impact of emotion and government trust on individuals' risk information seeking and avoidance during the COVID-19 pandemic: a cross-country comparison. J Health Commun.

[66] Erhardt J, Freitag M, Filsinger M, Wamsler S (2021). The emotional foundations of political support: how fear and anger affect trust in the government in times of the covid-19 pandemic. Schweiz Z Polit.

[67] Khalifa H (2020). COVID-19 pandemic and trust in government: the case of Bahrain. Int J Manag.

[68] Bobkowski PS, Shafer A, Ortiz RR (2016). Sexual intensity of adolescents' online self-presentations: joint contribution of identity, media consumption, and extraversion. Comput Hum Behav.

[69] Shafer A, Bobkowski P, Brown JD (2013). Sexual media practice: how adolescents select, engage with, and are affected by sexual media. The Oxford Handbook of Media Psychology.

[70] Chen D, Cheng CY, Urpelainen J (2016). Support for renewable energy in China: a survey experiment with internet users. J Clean Prod.

[71] Huang Q (2019). Understanding public perceptions of genetically modified organisms in China: the role that heuristics play during digital media exposure. Chin J Commun.

[72] Lien CH, Cao Y (2014). Examining WeChat users’ motivations, trust, attitudes, and positive word-of-mouth: evidence from China. Comput Hum Behav.

[73] Sharma H (2022). How short or long should be a questionnaire for any research? Researchers dilemma in deciding the appropriate questionnaire length. Saudi J Anaesth.

[74] Yu W, Shen F (2021). Does fact-checking habit promote COVID-19 knowledge during the pandemic? Evidence from China. Public Health.

[75] Karnowski V, Kümpel AS, Leonhard L, Leiner DJ (2017). From incidental news exposure to news engagement. How perceptions of the news post and news usage patterns influence engagement with news articles encountered on Facebook. Comput Hum Behav.

[76] Kim HK, Niederdeppe J (2013). The role of emotional response during an H1N1 influenza pandemic on a college campus. J Public Relations Res.

[77] Mayer RC, Davis JH (1999). The effect of the performance appraisal system on trust for management: a field quasi-experiment. J Appl Psychol.

[78] Oeldorf-Hirsch A (2017). The role of engagement in learning from active and incidental news exposure on social media. Mass Commun Soc.

[79] Li Y, Wang X (2018). Seeking health information on social media: a perspective of trust, self-determination, and social support. J Organ End User Comput.

[80] Hayes AF (2013). Introduction to Mediation, Moderation, and Conditional Process Analysis: A Regression-Based Approach.

[81] Zhai Y (2022). Government policy performance and central–local political trust in China. J Public Policy.

[82] Chen D (2017). Local distrust and regime support: sources and effects of political trust in China. Pol Res Q.

[83] Yang Z, Xin Z (2019). Income inequality and interpersonal trust in China. Asian J Soc Psychol.

[84] Festinger L (1957). A Theory of Cognitive Dissonance.

[85] Harmon-Jones E, Mills J, Harmon-Jones E (2019). An introduction to cognitive dissonance theory and an overview of current perspectives on the theory. Cognitive Dissonance: Reexamining a Pivotal Theory in Psychology.

[86] Hamilton LC, Hartter J, Saito K (2015). Trust in scientists on climate change and vaccines. Sage Open.

[87] Liu P, Zhong X, Yu S (2020). Striking a balance between science and politics: understanding the risk-based policy-making process during the outbreak of COVID-19 epidemic in China. J Chin Gov.

[88] Ren F (2021). A reconsideration on telling good stories about Chinese scientists and shaping the exemplary image of scientists amid the anti-pandemic campaign. Cultur Sci.

[89] Rieger MO, Wang M (2022). Trust in government actions during the COVID-19 crisis. Soc Indic Res.

[90] Zhang T, Yu L (2021). The relationship between government information supply and public information demand in the early stage of COVID-19 in China-an empirical analysis. Healthcare (Basel).

[91] Hofstede G (2001). Culture′s Consequences: Comparing Values, Behaviors, Institutions and Organizations Across Nations.

[92] House RJ, Hanges PJ, Javidan M, Dorfman PW, Gupta V (2004). Culture, Leadership, and Organizations: The GLOBE Study of 62 Societies.

[93] Sztompka P, Sasaki M (2019). Trust in the moral space. Trust in Contemporary Society.

[94] Yang J, Dong C, Chen Y (2021). Government’s economic performance fosters trust in government in China: assessing the moderating effect of respect for authority. Soc Indic Res.

[95] He AJ, Shi Y, Liu H (2020). Crisis governance, Chinese style: distinctive features of China’s response to the Covid-19 pandemic. Policy Des Pract.

[96] Zhong K, Liu Y, Christensen T (2022). Crisis coordination in centralized regimes: explaining China's strategy for combatting the COVID-19 pandemic. Int Public Manag J.

[97] Lehdonvirta V, Oksanen A, Räsänen P, Blank G (2020). Social media, web, and panel surveys: using non‐probability samples in social and policy research. Policy Internet.

